# Neuroinflammation after stroke: initiation, amplification and therapeutic prospects

**DOI:** 10.1186/s12967-026-08103-3

**Published:** 2026-04-11

**Authors:** Yushuo Duan, Liudan Yao, Xianyang Liang, Yingda Xie, Xinyu Gu, Ruile Shen

**Affiliations:** 1 Department of Neurology, The First Affiliated Hospital, College of Clinical Medicine, Henan University of Science and Technology, Luoyang, Henan 471003 China; 2 School of Basic Medical Sciences, Henan University of Science and Technology, Luoyang, Henan 471003 China; 3 Henan Key Laboratory of Cancer Epigenetics, Cancer Institute, The First Affiliated Hospital, College of Clinical Medicine, Henan University of Science and Technology, Luoyang, Henan 471003 China

**Keywords:** Stroke, Neuroinflammation, Initiation, Amplification, Treatment strategies

## Abstract

**Background:**

Stroke is one of the leading causes of death and long-term disability worldwide, placing a significant health and economic burden on society. Despite significant advancements in acute reperfusion therapy, the narrow treatment window makes it challenging for many patients to get the help they need. The pathophysiological core of post-stroke brain injury lies in the cascade of neuroinflammation amplification initiated by the activation of innate immune cells that release a variety of inflammatory mediators, forming a positive feedback loop that keeps amplifying inflammatory signals. This uncontrolled and self-sustaining excessive inflammation, a major driver of secondary neuronal injury, also contributes to neurological deficits. Therefore, it is crucial to understand how neuroinflammation is initiated.

**Objective:**

This review aims to systematically elucidate the initiation mechanisms and cascade amplification effects of neuroinflammation after stroke, revealing how glial cell metabolic reprogramming triggered by damage-associated molecular patterns (DAMPs) drives blood-brain barrier (BBB) disruption and peripheral immune cell infiltration. It also focuses on the interactive crosstalk between inflammation and various cell death pathways, analyzing the molecular mechanisms that form a vicious cycle and exacerbate secondary brain injury. Furthermore, by reviewing existing intervention strategies targeting neuroinflammation, this paper discusses clinical translation barriers such as patient heterogeneity and drug delivery efficiency, with the goal of providing a theoretical foundation and strategic reference for the precise intervention of post-stroke neuroinflammation.

**Conclusion:**

In the future, as single-cell sequencing and multi-omics analysis techniques become more widely available, researchers will be able to more systematically clarify the spatiotemporal dynamics and individual heterogeneity of neuroinflammation mechanisms. The findings included in this review could make a difference in moving relevant basic research into clinical applications and offer important insights for developing new, precise and effective targeted treatments.

## Introduction

Stroke is a serious threat to human health and also one of the main causes of disability, with high rates of both incidence and mortality [1]. On the basis of its pathological mechanisms, stroke is primarily divided into two types: ischemic and hemorrhagic, accounting for about 85% and 15%, respectively. Although significant progress has been made in early pharmacological thrombolysis and endovascular thrombectomy, the narrow therapeutic time window severely limits access to treatment, with only a small percentage of patients benefiting from these time-sensitive therapies [2, 3]. Therefore, elucidating the pathophysiological mechanisms of post-stroke neuronal injury is essential for the development of more effective therapeutic strategies.

Traditional views suggest that neuronal excitotoxicity and oxidative stress are the main causes of acute injury following cerebral ischemia [4, 5]. However, recent studies indicate that neuroinflammation, which follows the primary injury, is a key factor driving secondary damage to brain tissue, increasing the size of the infarct, and worsening neurological function [6–8].

The initiation of neuroinflammation is not simply a passive reaction but rather a highly ordered cascade effect actively triggered by endogenous danger signals. When stroke occurs, damaged or necrotic neurons and glial cells quickly release many damage-associated molecular patterns (DAMPs) [6]. These endogenous alarm signals can be recognized by pattern recognition receptors (PRRs) on resident immune cells in the brain, as well as by peripheral immune cells that infiltrate the brain parenchyma, activating downstream key inflammatory signaling pathways and resulting in the release of large amounts of pro-inflammatory cytokines and chemokines, forming a “cytokine storm” [9]. This further disrupts the structural and functional integrity of the blood-brain barrier (BBB), promoting the chemotaxis and subsequent infiltration of more peripheral immune cells to the lesion area, exacerbating the inflammatory response in the brain parenchyma and creating a vicious cycle that feeds on itself and ultimately culminates in significant dysfunction and even the complete collapse of neural network activity. Therefore, targeting the critical molecular events in this initiation process may block the subsequent cascade amplification, thereby providing new therapeutic strategies for neuroprotection after stroke.

This review constructs a logical framework for the progression of neuroinflammation from initial trigger to cascade amplification, focusing on key processes such as the interaction between DAMPs and PRRs, glial cell polarization, and immune cell migration, as well as looking at inflammation signaling pathways linked to cell death and metabolism. Furthermore, the review evaluates therapeutic strategies targeting the initiation phase and discusses the challenges associated with their clinical translation, with the goal of identifying novel approaches to improve long-term outcomes.

## Initiation of neuroinflammation: triggering of central innate immunity

### Release of danger signal molecules

The initiation of neuroinflammation upon a stroke event is a well-organized cascade process actively triggered by endogenous danger signals. During the acute phase of stroke, ischemia and hypoxia cause the death or damage of neurons and glial cells in both the ischemic core and the penumbra. These injured cells release various DAMPs that act as the initial triggers for neuroinflammation, such as adenosine triphosphate (ATP), high mobility group box 1 (HMGB1), heat shock proteins (HSPs), fragmented DNA/RNA segments, and leaked mitochondrial DNA (mtDNA) [10].

The DAMPs released by injured cells are subsequently recognized by resident immune cells in the brain (such as microglia) and peripheral immune cells infiltrating into the brain tissue via PRRs, thereby activating downstream key inflammatory signaling pathways and promoting the release of cytokines [11]. Specifically, extracellular ATP binds to purinergic receptors (P2 × 7R/P2Y2R) on microglia and astrocytes, opening cation channels, causing mitochondrial dysfunction and lysosomal rupture, and triggering the assembly of the NLRP3 inflammasome. The activated inflammasome cleaves and activates caspase-1, promoting the maturation and release of interleukin-1beta (IL-1β) and IL-18 while also inducing a form of programmed cell death (PCD) known as pyroptosis. After pyroptotic cells rupture, they release DAMPs once more, creating a self-reinforcing feedback loop that dramatically amplifies the inflammatory response [12].

Correspondingly, HMGB1 and other DAMPs mainly act via receptors such as Toll-like receptors (TLRs) and the receptor for advanced glycation end products (RAGE). HMGB1 is recognized by TLR2/4, activating the MyD88-dependent nuclear factor kappaB (NF-κB) pathway. Activation of NF-κB, a core pathway mediating the transcription of inflammatory mediators, drives the massive expression of key pro-inflammatory factors such as tumor necrosis factor-alpha (TNF-α), IL-1β, and IL-6. These factors break down the tight junctions of the BBB, increasing its permeability and encouraging more immune cells and inflammatory mediators to infiltrate the brain tissue, ultimately resulting in the spread of inflammation to surrounding brain regions [13]. Additionally, HMGB1 can also bind to RAGE, persistently activating the mitogen-activated protein kinase (MAPK) and phosphatidylinositol 3-kinase (PI3K)-protein kinase B (Akt)-NF-κB axis. Among these pathways, the MAPK pathway, as another core signaling cascade, primarily responds to stress signals and mediates the release of immediate early inflammatory factors, exhibiting temporal synergy and complementarity with NF-κB. This signaling axis drives massive infiltration of peripheral neutrophils and monocytes/macrophages into the brain tissue. The mediators released by these cells, such as proteases and reactive oxygen species (ROS), further amplify the inflammatory signals and directly exacerbate tissue damage [14, 15].

In summary, the interaction between DAMPs and PRRs is a central element that initiates and drives the vicious cycle of neuroinflammation after a stroke, which involves cell death, immune activation and BBB disruption, and is a major cause of neurological damage and functional deficits after a stroke. However, given that DAMPs also participate in tissue repair under physiological conditions, achieving precisely targeted intervention without disrupting their baseline physiological functions remains a major challenge in clinical translation efforts.

### Immediate response of glial cells

Microglia, as the resident immune cells of the central nervous system, are core participants in neuroinflammation following stroke, and the dynamic balance of their phenotypic polarization (M1/M2) profoundly influences the outcomes of neural injury and repair. After stroke, at the core of the injury, the massive release of DAMPs rapidly drives microglia to polarize into the pro-inflammatory M1 phenotype, releasing mediators such as TNF-α, IL-1β, ROS, and reactive nitrogen species (RNS), inducing neuronal injury and BBB disruption [16]. It is noteworthy that their core function, namely the phagocytosis and clearance of cellular debris, exerts a protective effect. However, this function also involves the release of ROS, and this process may further amplify inflammation and exacerbate surrounding tissue injury [17]. This underscores the inherent concomitant relationship between the protective functions and damaging effects of microglia. In contrast, in the peri-infarct area, some microglia can polarize into the M2 phenotype, secreting IL-10, TGF-β and other anti-inflammatory factors, and producing neurotrophic factors like brain-derived neurotrophic factor (BDNF) and glial cell line-derived neurotrophic factor (GDNF) that aid in repair [18, 19]. Therefore, switching to the M2 phenotype after a stroke helps resolve inflammation, modulate glial scar formation, and promote tissue repair, whereas prolonged retention in the M1 state can result in chronic inflammation, severely hindering the recovery of neural function [20]. This dynamic imbalance represents the critical tipping point at which the “dual role” of microglia shifts from a protective to a damaging function. Accurately targeting the timing of this polarization is a key goal for future therapeutic strategies.

A1 reactive astrocytes profoundly affect the inflammatory process and tissue health after stroke. They are induced by factors such as IL-1α, TNF-α and C1q released from M1 phenotype microglia, which not only release chemokines such as C-C motif chemokine ligand 2 (CCL2) and C-X-C motif chemokine ligand 10 (CXCL10) and pro-inflammatory factors that worsen neuroinflammation [21, 22] but also, by releasing matrix metalloproteinases (MMPs), disrupt the integrity of the BBB, promoting the infiltration of peripheral immune cells and further worsening the inflammatory response [23]. The A2 phenotype helps reduce secondary damage from oxidative stress by boosting glutathione levels [24]. At the same time, this phenotype secretes factors such as angiopoietin-1 (Ang-1) and BDNF, enhancing BBB integrity and promoting BBB repair [25]. The bidirectional regulatory interaction between astrocytes and microglia is particularly crucial: activated A1 astrocytes send feedback that promotes the M1 polarization of microglia through signaling pathways such as C3/C3aR, forming an inflammatory amplification loop that continuously drives tissue damage [26]. Therefore, intervening in this cycle is key to managing neuroinflammation after stroke.

In summary, the complex interaction between microglia and astrocytes after stroke establishes a positive feedback loop of inflammation that is highly dependent on energy metabolism. Targeting key signaling nodes within this circuit offers a promising approach to fundamentally block the cascade amplification of neuroinflammation, thereby providing emerging therapeutic targets for promoting functional recovery after stroke.

## Amplification of neuroinflammation: BBB disruption and peripheral immune recruitment

### Oxidative stress and activation of MMPs

After a stroke occurs, local ischemia and hypoxia induce mitochondrial damage, resulting in the excessive production of ROS. During the ischemia-reperfusion process, NADPH oxidase (NOX), xanthine oxidase (XO) and others are activated, further exacerbating ROS production [27]. This surge in ROS level is a key factor in the destruction of the BBB and the amplification of neuroinflammation. First, ROS directly oxidize claudin-5, a tight junction protein, and induce endothelial cell apoptosis, directly damaging the structural integrity of the BBB [28]. Second, ROS function as key signaling molecules, activating downstream signaling pathways. On the one hand, ROS activate the NF-κB pathway, promoting the overproduction of key pro-inflammatory factors such as TNF-α, IL-1β, and IL-6, thereby exacerbating inflammation [29]; on the other hand, they activate the ASK1-JNK pathway [30] and directly oxidize key necroptotic proteins such as RIPK1/RIPK3, thereby regulating various forms of cell death, such as apoptosis and necroptosis [31]. Ultimately, these cell deaths triggered by ROS will release DAMPs once more, reactivating the immune response. This surge in ROS then forms a self-amplifying vicious cycle with inflammatory signals and cell death, continuously aiding the pathological progression following a stroke.

In this process, the activation of MMPs is a key step in converting inflammatory signals into structural damage. Following stroke, both cytokines and ROS, released by the inflammatory response, can upregulate the expression and activity of MMP-2 and MMP-9. Activated MMPs target and degrade the basement membrane and tight junction proteins of the BBB, compromising its structural integrity [32]. The destruction of the BBB directly facilitates the infiltration of peripheral immune cells and greatly raises the risk of hemorrhagic transformation [33]. These infiltrating cells further release ROS and inflammatory factors, creating a self-reinforcing cycle with MMPs, ultimately spreading the damage from local ischemic foci to a full-body inflammatory storm.

The role of MMPs is characterized by a significant dual role. Although early activation of MMP-9 mediates BBB disruption and the secondary cerebral edema, recent studies have shown that MMP-9 also participates in neurovascular unit remodeling, promotes angiogenesis, and enhances synaptic plasticity during the subacute phase of stroke [34]. Therefore, therapeutic strategies targeting MMPs should carefully consider the therapeutic time window to avoid inhibiting their beneficial functions during the repair phase.

### Peripheral immune cell infiltration

After a stroke event, the initiation of neuroinflammation not only involves the activation of central innate immune cells but also relies on the ongoing infiltration of peripheral immune cells [35]. Specifically, ischemic or hemorrhagic brain injury directly disrupts the integrity of the BBB. At the same time, damaged neurons and endothelial cells extensively release DAMPs, which activate microglia and astrocytes. These activated glial cells in turn release pro-inflammatory cytokines and chemokines, further worsening BBB dysfunction and letting peripheral immune cells cross the barrier and get into the brain tissue [33, 36]. This process is carefully controlled by the chemical gradient formed by the chemokines released from the ischemic core and the peri-infarct area.

#### Neutrophils: amplifiers of acute injury

Neutrophils, as the first peripheral immune cells that arrive in ischemic brain tissue, rapidly cross the damaged BBB mediated by chemokines such as CXCL1/CXCL2 and CCL3 [37, 38]. They not only damage tissues directly by releasing substances like ROS and MMPs but can also trigger neutrophil extracellular traps (NETs), releasing NETs of chromatin and granule proteins. While NETs capture pathogens, they can also serve as DAMPs that directly activate TLRs, inducing necroptosis and apoptosis in endothelial cells, amplifying local inflammation, and further exacerbating brain ischemic injury through promoting microvascular thrombosis [39].

#### Monocytes/macrophages: infiltration and phenotypic polarization

The migration of monocytes/macrophages to the ischemic brain region is primarily driven by the CCL2/CCR2 chemotactic axis. After a stroke, damaged neurons, astrocytes and endothelial cells express high levels of CCL2, which guides circulating monocytes to cross the BBB and infiltrate the brain parenchyma by binding to CCR2 receptors on their surface, and then differentiate into macrophages [36]. Once in the brain parenchyma, they differentiate into pro-inflammatory (M1) or anti-inflammatory (M2) phenotypes depending on signals from the microenvironment. The dynamic balance between these two phenotypes directly influences the progression of neuroinflammation and the outcome of tissue repair, a key factor in stroke prognosis [40].

#### T lymphocyte: bridging innate and adaptive immunity

T lymphocyte infiltration in stroke shows functional duality and high spatiotemporal heterogeneity. In this context, the balance of CD4⁺ T cell types is key to managing neuroinflammation. Specifically, effector T cells (such as Th1 and Th17) cause delayed inflammatory damage in the late phase of ischemia by releasing pro-inflammatory factors like IFN-γ, TNF-α and IL-17, which worsen neuroinflammation and neuronal apoptosis [19]. On the other hand, Th2 cells and regulatory T cells (Treg) reduce the activity of effector T cells by secreting cytokines such as IL-4, IL-5, TGF-β and IL-10, and encourage microglia to change into the M2 type, thereby providing neuroprotective and tissue repair effects [41]. Importantly, the protective effect of Treg cells depends on timing: early suppression may worsen the inflammatory response, resulting in a worsening of neurological function, while too much suppression later on can interfere with important immune regulation and tissue repair. Therefore, boosting Treg cell function at the right time could be a promising strategy to improve the long-term recovery of neurological function after stroke.

**Fig. 1:**
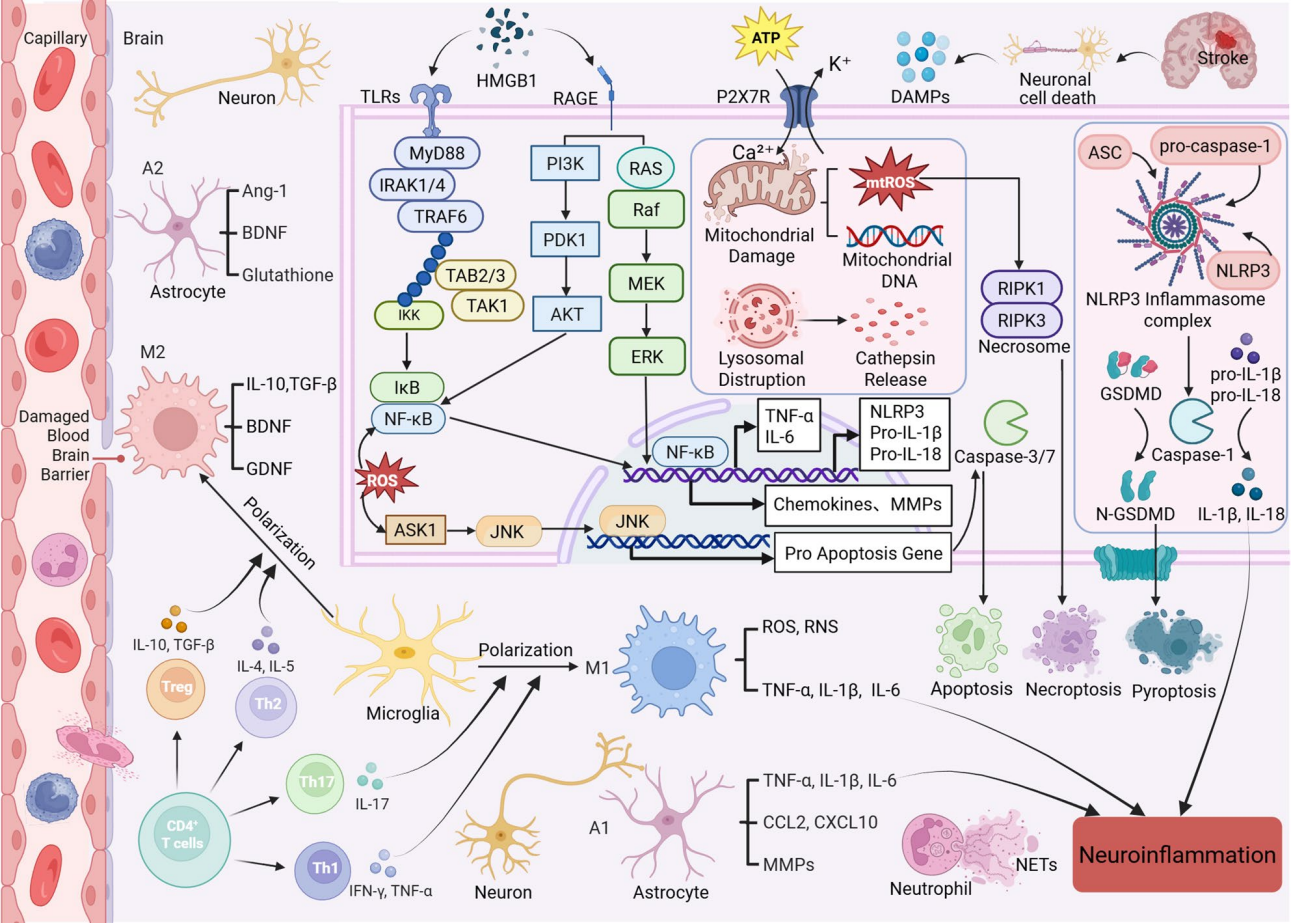
Overview of the initiation and amplification mechanisms of neuroinflammation after stroke. Stroke triggers brain cells (neurons and astrocytes) to release damage-associated molecular patterns (DAMPs) like HMGB1 and ATP; these danger signals turn on receptors like TLR4/2, RAGE and P2 × 7R on the microvascular endothelium, initiating the MyD88-TRAF6-TAK1-NF-κB and NLRP3 inflammasome pathways, which results in an excessive release of IL-1β, TNF-α, IL-6, chemokines, and MMPs. The neutrophils and CD4⁺ T cells recruited in this process trigger M1-type microglial activation, causing damage to the blood-brain barrier (BBB), breaking down the extracellular matrix, and resulting in more neuronal death, creating a vicious cycle of injury, inflammation, and further nerve injury

In summary, peripheral immune cells are recruited to the brain parenchyma through the precise guidance of chemotactic gradients. The temporal infiltration and phenotypic switch are closely linked to the ultimate direction of neuroinflammation: either a shift toward reparative regeneration or progression into the vicious cycle of chronic inflammation. The core of future treatment lies in decoding chemotactic signals and modulating the functional remodeling of various cell subpopulations. Achieving this goal requires careful consideration of patient heterogeneity and precise timing of interventions (Fig. 1).

## Amplification and maintenance of neuroinflammation

### Metabolic reprogramming of immune cells

In the acute phase of stroke, the functional status of glial cells is closely associated with their metabolic pathways. The ischemic microenvironment triggers an increase in glycolytic activity, disturbances in lipid metabolism, mitochondrial damage, and abnormal accumulation of amino acids that together form a metabolic network driving the early inflammatory cascade.

#### Glycolysis

**Fig. 2:**
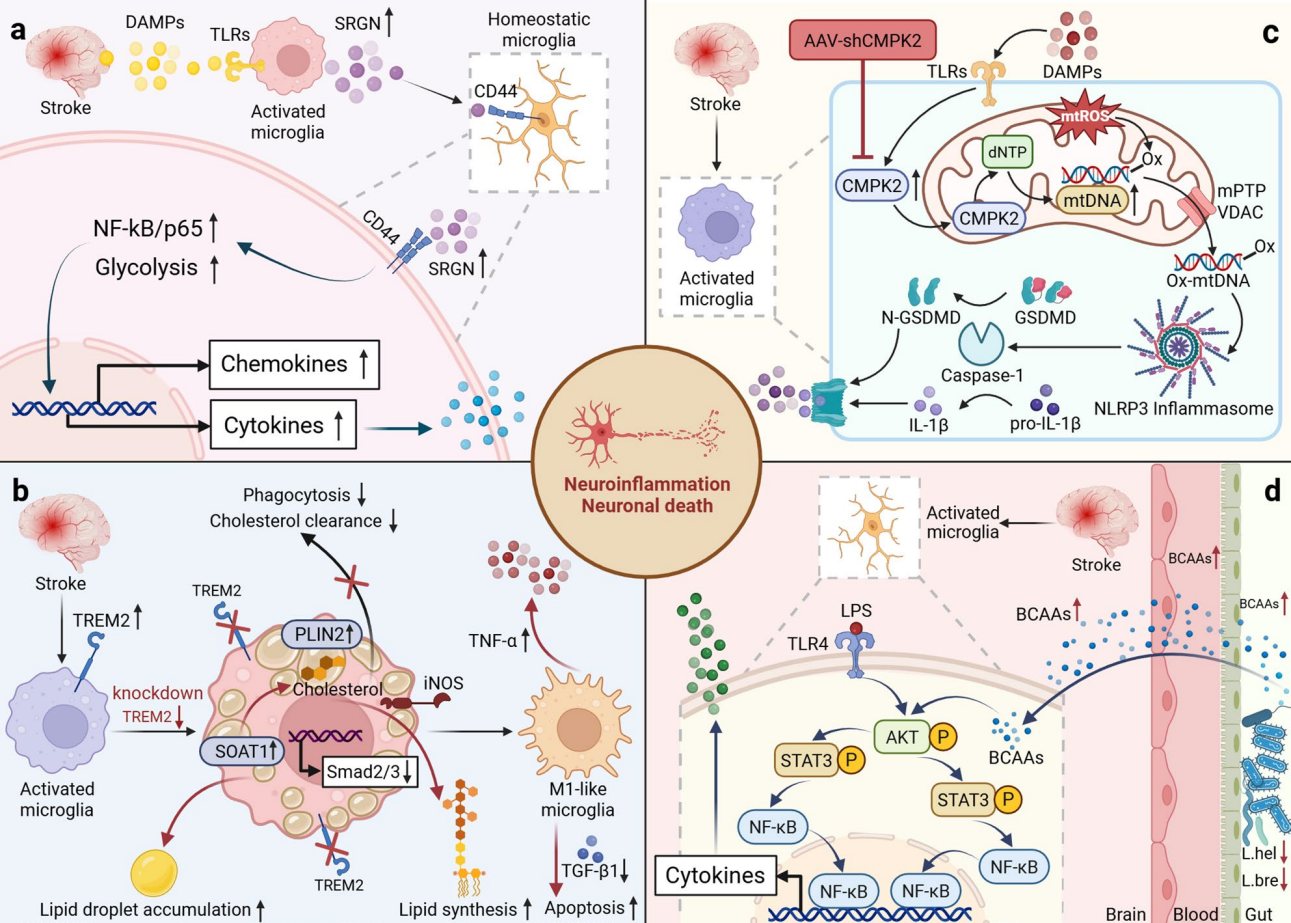
Bidirectional regulation model of “cell metabolism-neuroinflammation” after stroke. (**a**) **Glycolysis**: DAMPs upregulate SRGN via TLRs, then SRGN activates the NF-kB p65 signaling pathway by binding to the CD44 receptor, boosting glycolysis in microglia, promoting the release of pro-inflammatory cytokines, and exacerbating neuroinflammation. (**b**) **Lipid metabolism**: Downregulation of TREM2 can inhibit the TGF-β1/Smad2/3 signaling pathway and upregulate the expression of SOAT1 and PLIN2, causing microglia to have impaired phagocytosis, lipid droplet accumulation, and hindered M2 polarization, finally resulting in the sustained secretion of pro-inflammatory factors such as iNOS and TNF-α, aggravating neuroinflammation. (**c**) **Mitochondrial dysfunction**: After stroke, mitochondrial ROS increase, mPTP stays wide open, DAMPs upregulate CMPK2 via TLRs, thus accelerating dNTP synthesis, promoting the synthesis and release of Ox-mtDNA, which then kicks off NLRP3 inflammasome assembly, triggering Caspase-1 cleavage of GSDMD and mediating IL-1β maturation, ultimately amplifying neuroinflammation and inducing neuronal pyroptosis. (**d**) **Amino acid metabolism**: The reduction of gut colonization by *Lactobacillus helveticus* (L.hel) and *Lactobacillus brevis* (L.bre) causes an unexplained buildup of BCAA, that activates the Akt/STAT3/NF-κB signaling pathway along the gut-brain axis, promoting the release of pro-inflammatory factors and worsening neuroinflammation in microglia

The sharp decline in cerebral blood flow after stroke compels cells to shift rapidly to anaerobic glycolysis, resulting in substantial lactate accumulation and local tissue acidosis. This acidic microenvironment not only directly aggravates neuronal metabolic impairment but also acts as an endogenous danger signal, rapidly activating the inflammatory transcriptional program of microglial cells. Mechanistic studies have shown that the early upregulation of Serglycin (SRGN) during ischemia can activate the NF-κB p65 pathway by binding to the CD44 receptor, promoting glycolysis in microglial cells and thereby exacerbating neuroinflammation [42] (Fig. 2a). Chemokine-like factor 1 (CKLF1) can also mediate similar metabolic reprogramming through the AMPK-mTOR-HIF-1α pathway [43].

In terms of clinical translation, neuroimaging studies based on 18 F-FDG PET have revealed altered glucose metabolism in the penumbra region of patients with acute ischemic stroke (AIS) [44]. Similarly, multiple clinical studies have reported a sharp increase in peripheral blood lactate levels in patients during the acute phase. This increase not only provides direct evidence of intensified anaerobic glycolysis in the central nervous system but is also significantly associated with more pronounced systemic inflammatory responses, prolonged hospital stays, and higher short-term mortality [45, 46]. However, targeting glycolysis presents a classic “double-edged sword” dilemma: compensatory glycolysis in the very early stages of ischemia is crucial for maintaining neuronal cell survival, while sustained glycolysis drives the worsening of inflammation. This suggests that clinical interventions targeting glycolysis require exceptionally precise temporal control.

#### Lipid metabolism

Lipids are not only structural components of the cell membrane but also direct signaling molecules that regulate the early activation of glial cells [47]. In the initial stage of stroke, lipid metabolic reprogramming in glial cells serves as a core driver of inflammatory cascade amplification. At the mechanistic level, downregulation of triggering receptor expressed on myeloid cells 2 (TREM2) during the acute ischemic phase inhibits the TGF-β1/Smad2/3 signaling pathway, resulting in impaired phagocytic function in microglia and disruption of lipid homeostasis. This early metabolic reprogramming triggered by TREM2 downregulation not only induces subsequent pathological accumulation of lipid droplets but also ultimately blocks the polarization of microglia toward a neuroprotective phenotype, thereby driving a downstream vicious cycle of inflammation [48] (Fig. 2b). Additionally, early dysregulation of signaling crosstalk between astrocytes and microglia (particularly the VEGFD/VEGFR3 axis) hinders glycerophospholipid synthesis, similarly inducing pathological accumulation of lipid droplets. This lipid remodeling process has been identified as a key metabolic checkpoint that drives microglia to undergo early pro-inflammatory transformation and maintain a long-term inflammatory state [49].

Clinical studies have found that plasma levels of long-chain ceramides are significantly elevated in patients with AIS, and mechanistic research further confirms that they exacerbate neuroinflammation after ischemic brain injury by activating the cGAS-STING pathway [50]. However, the high heterogeneity of clinical patients presents a significant translational barrier: stroke populations often have underlying metabolic diseases such as hyperlipidemia and diabetes mellitus, and this systemic baseline lipid disorder may significantly amplify the primary inflammatory response in the central nervous system. Therefore, in the development of drugs targeting lipid metabolism, careful consideration must be given to individual differences and the practical challenges posed by comorbidities.

#### Mitochondrial dysfunction

Mitochondria are the core organelles of cellular energy metabolism and are crucial for maintaining energy homeostasis in brain tissue. During cerebral ischemia, hypoxia directly results in the cessation of mitochondrial oxidative phosphorylation, further inducing oxidative stress, impairing mitochondrial membrane integrity, and damaging mtDNA, ultimately resulting in the widespread disruption of cellular metabolism and forming a vicious cycle of energy depletion and increased oxidative damage. Recent studies have found that after stroke, in astrocytes, ERK/p38 mediates the phosphorylation of peroxiredoxin6 (PRDX6) at the Thr177 site, boosting the activity of calcium-independent phospholipase A2 (iPLA2), which then triggers ROS production by increasing NOX2 and the Drp1-mitochondrial fission pathway, ultimately pushing microglia/macrophages to shift towards the pro-inflammatory M1 phenotype, significantly amplifying the neuroinflammatory cascade [51]. Research by Kong et al. showed that mtDNA is released into the cytoplasm of microglia under ischemia/reperfusion (I/R) injury, activating STING-mediated IRF3/NF-κB signaling to promote microglial polarization to the M1 phenotype, ultimately exacerbating neuroinflammation and ischemic injury [52].

On the other hand, regarding the protective mechanisms, studies have found that knocking down cytidine/uridine monophosphate kinase 2 (CMPK2) can effectively stop the formation of oxidized mtDNA (Ox-mtDNA), thereby preventing the activation of NLRP3 inflammasome in microglia/macrophages, lessening neuroinflammation and ischemic injury [53] (Fig. 2c). Similarly, promoting mitochondrial autophagy for the clearance of damaged mitochondria has been demonstrated to effectively interrupt this source of inflammatory amplification [54]. Similar to glycolysis, mitochondrial stress exerts dual effects: moderate stress can initiate protective autophagy, while excessive mitochondrial disintegration pushes cells toward an irreversible death pathway. Therefore, future mitochondrial-targeted therapies must carefully balance mitigating inflammatory damage and preserving endogenous protective mechanisms.

#### Amino acid metabolism

The massive release of excitotoxic glutamate is a hallmark event in the initial stages of cerebral ischemia. In addition to directly causing neuronal injury, glutamate initiates dual injury through postsynaptic density protein 93 (PSD-93): first, it mediates excitotoxicity; second, it binds to C-X3-C motif chemokine ligand 1 (CX3CL1) to activate microglia, triggering neuroinflammation. These two processes act synergistically, thereby amplifying ischemic brain injury [55]. Furthermore, the significant enrichment of branched-chain amino acid (BCAA) in ischemic brain tissue can act as metabolic signals to directly drive microglia-mediated neuroinflammation by activating the Akt/STAT3/NF-κB signaling pathway [56] (Fig. 2d).

In clinical translation, intervention strategies targeting amino acid metabolism have shown preliminary neuroprotective potential. The traditional Chinese medicine preparation Danhong injection (DHI) has been shown to promote microglial polarization toward an anti-inflammatory phenotype by remodeling their arginine metabolic pathway [57]. Additionally, gut microbiota components (such as *Lactobacillus helveticus* and *Lactobacillus brevis*) can exert neuroprotective effects by reducing systemic BCAA levels [56]. This suggests that the gut microbiota–amino acid metabolism–neuroinflammation axis may become a new intervention target. However, the amino acid metabolism network is highly intricate, and the same amino acid often plays completely opposite roles in different pathological stages and across different cell populations. In the future, there is an urgent need to systematically decode the spatiotemporal heterogeneity of amino acid metabolism in post-stroke neuroinflammation using spatial multi-omics technologies.

In summary, glycolysis-driven lactate accumulation and inflammatory activation; ceramide accumulation mediated by lipid metabolism disorders and cGAS-STING pathway activation; mtDNA release triggered by mitochondrial damage and NLRP3 inflammasome initiation; and excitotoxicity mediated by abnormal amino acid metabolism collectively form a multidimensional evidence chain within the metabolic reprogramming network that drives neuroinflammation. This metabolic reprogramming not only provides energy support for the phenotypic transformation of glial cells but also serves as the core driver of inflammatory cascade amplification by enhancing local inflammatory signals and promoting peripheral immune cell recruitment.

### Cross-talk in cell death regulation

After stroke, the irreversible necrosis in the ischemic core rapidly releases DAMPs and ROS. These early signals not only directly damage adjacent cells but also initiate a complex network of PCD in the penumbra region [58]. Typical forms of PCD, such as autophagy and pyroptosis, are active cellular processes regulated by specific molecular signaling pathways. These processes share key upstream regulatory nodes, which collectively modulate the fate of nerve cells and the outcome of inflammation.

#### Autophagy

Autophagy, as the primary degradation pathway within cells, plays a critical dual role in the pathology of stroke. Moderate autophagy exerts neuroprotective effects through the clearance of damaged mitochondria and inhibition of excessive NLRP3 inflammasome activation [58]; however, excessive autophagic flux may induce cell death [59].

**Fig. 3:**
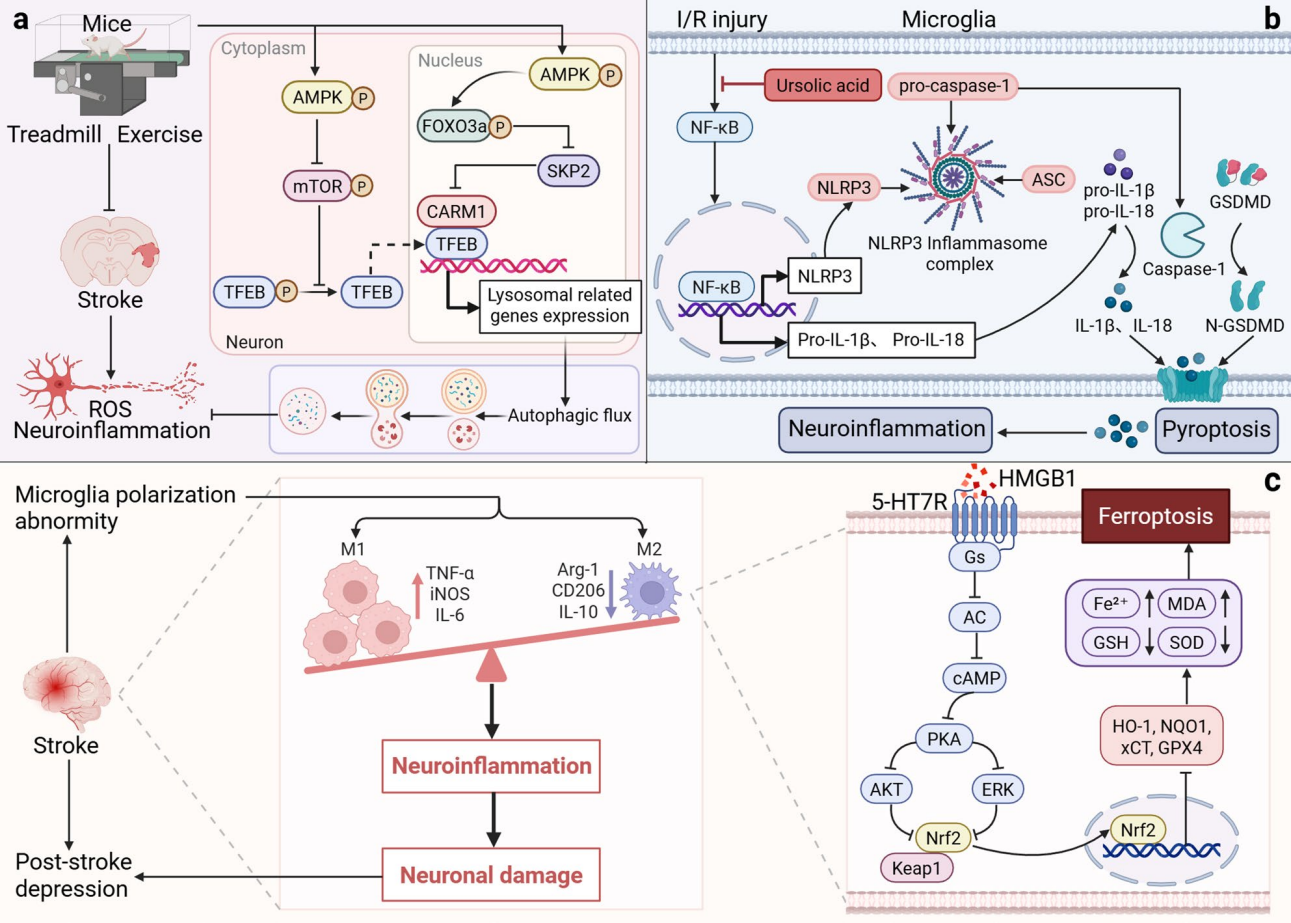
Bidirectional regulation model of “cell death-neuroinflammation” after stroke. (**a**) **Autophagy**: Exercise preconditioning activates transcription factor EB (TFEB) and regulates the AMPK-mTOR and AMPK-FOXO3a-SKP2-CARM1 signaling pathways, driving both lysosomal and autophagic flux. This process inhibits neuroinflammation and oxidative stress, ultimately providing neuroprotection. (**b**) **Pyroptosis**: Cerebral ischemia-reperfusion injury activates NF-κB nuclear translocation, initiating NLRP3 inflammasome assembly and activating Caspase-1. This results in the release of IL-1β/IL-18 and GSDMD-mediated neuronal pyroptosis, making neuroinflammation worse. Ursolic acid (UA) effectively inhibits NF-κB nuclear translocation, blocking this critical initiating signal and providing neuroprotection. (**c**) **Ferroptosis**: HMGB1 inhibits the cAMP/PKA and Nrf2/xCT/GPX4 pathways, causing a buildup of Fe²⁺and MDA, as well as the depletion of GSH and SOD. This induces ferroptosis in M2 microglia, exacerbating neuronal injury and resulting in symptoms similar to depression

The AMPK-mTOR signaling axis is the central regulator of this balance. Studies have confirmed that both pharmacological interventions (such as polyphyllin I [60] and β-elemene [61]) and non-pharmacological approaches (such as exercise pretreatment [62]) can restore impaired autophagic flux. Although these interventions differ in their regulatory mechanisms, they all promote microglial polarization from the pro-inflammatory M1 phenotype to the anti-inflammatory M2 phenotype, thereby blocking the inflammatory cascade at its source. For example, exercise pretreatment improves autophagic flux and alleviates oxidative stress and inflammatory responses by activating transcription factor EB (TFEB) and regulating the AMPK-mTOR and AMPK-FOXO3a-SKP2-CARM1 signaling pathways [62] (Fig. 3a).

#### Pyroptosis

Unlike the protective clearance of autophagy, pyroptosis is a pro-inflammatory form of cell death characterized by the formation of plasma membrane pores and the explosive release of inflammatory factors (IL-1β, IL-18). It serves as a key execution step in the vicious cycle of neuroinflammation following stroke [63]. The NLRP3 inflammasome acts as a molecular hub connecting ischemic injury to pyroptotic activation. On one hand, upon activation, NLRP3 mediates the cleavage of GSDMD via caspase-1, resulting in membrane pore formation; on the other hand, it promotes its own transcription and that of pro-inflammatory mediators through the NF-κB pathway, forming a positive feedback amplification loop that continuously exacerbates the inflammatory cascade.

The activation of NLRP3 is regulated by multiple upstream signals, among which the STING pathway, a key DNA damage sensor, plays a crucial initiating role in cerebral ischemia-reperfusion injury [64]. These upstream signals converge on the NF-κB/NLRP3 axis, making it a core target for pyroptosis intervention. Multiple studies have revealed that targeting this axis is an effective strategy to block the vicious cycle. For example, interventions including the natural component ursolic acid [65] (Fig. 3b), melatonin [66], sweet tea [67], and repetitive transcranial magnetic stimulation (rTMS) [68] all reduce GSDMD-mediated membrane rupture by inhibiting the overactivation of this axis, thereby blocking the secondary release of DAMPs and the spread of the inflammatory storm. This indicates that inhibiting pyroptosis not only protects individual cells but also plays a key role in interrupting the intercellular amplification of the inflammatory cascade.

#### Ferroptosis

Ferroptosis, an iron-dependent form of cell death driven by lipid peroxidation, is rapidly activated during the acute phase after stroke. It not only constitutes the initial event of neuronal injury but also drives the chronic continuation of neuroinflammation by releasing damage signals, thus being regarded as the “molecular switch” connecting acute injury to chronic pathology [69]. Its core characteristics are the depletion of glutathione peroxidase 4 (GPX4) activity and the lethal accumulation of lipid ROS, a process tightly monitored by the Nrf2 antioxidant system. There is a significant bidirectional promotion mechanism between ferroptosis and inflammation: on one hand, specific signals in the inflammatory microenvironment (such as HMGB1) can induce ferroptosis in M2-type microglia by inhibiting the Nrf2/GPX4 defense system, thereby compromising their neuroprotective function [70] (Fig. 3c). On the other hand, lipid peroxides released by ferroptotic cells act as potent DAMPs, further exacerbating tissue damage by activating inflammatory pathways in microglia [71].

In recent years, exosome-mediated intercellular communication has emerged as an important regulatory layer in this interactive network. Studies have found that macrophage-derived exosomes can target the endothelial cell OTUD5-GPX4 axis by carrying thrombospondin-1 (THBS1) to induce ferroptosis, disrupt BBB integrity, and indirectly activate glial cells to amplify neuroinflammation [72]. Meanwhile, foam cell-derived exosomal miRNA Novel-3 can directly induce ferroptosis in microglia by targeting the STRN-PI3K-Akt-mTOR axis, exacerbating neuroinflammation and brain injury [73]. These findings indicate that, although the regulatory mechanisms are diverse, ferroptosis-related signals ultimately converge on the core nodes of GPX4 activity and lipid peroxidation. Nrf2, as an upstream transcription factor regulating GPX4 and various antioxidant genes, is regarded as a common defense line against ferroptosis and neuroinflammation. Various natural drugs, such as caffeic acid [74], Icariside II [75], and neutral polysaccharide from Gastrodia elata (NPGE) [76], enhance cellular tolerance to oxidative stress by activating the Nrf2 axis, thereby simultaneously inhibiting ferroptosis and the accompanying inflammatory response.

#### Integrative mechanisms of cross-talk

Autophagy, pyroptosis, and ferroptosis are not independent events during stroke progression; rather, they are closely interconnected through ROS accumulation, mitochondrial damage, and the NF-κB/NLRP3 inflammatory axis. Autophagy is initially activated by the metabolic crisis triggered by ischemia to maintain cellular homeostasis. However, when this process is either insufficient or inhibited, the accumulated ROS and DAMPs trigger NLRP3-mediated pyroptosis and lipid peroxidation-driven ferroptosis. Collectively, these three cell death pathways form a dynamic inflammatory and cell-death interaction network.

The core nodes of this interaction network include the following aspects: First, ROS are the common upstream driving signal. Excessive ROS generated by mitochondrial dysfunction can both trigger NLRP3 inflammasome activation that mediates pyroptosis, and attack polyunsaturated fatty acids in cell membranes, inducing ferroptosis. Second, mitochondrial damage is the structural basis for the interaction. Damaged mitochondria not only activate the pyroptosis pathway by releasing DAMPs, but their dysfunction also directly compromises the cell’s defense against ferroptosis. Third, the NF-κB/NLRP3 inflammatory axis acts as a signal amplifier. Inflammatory factors such as IL-1β released during pyroptosis can further activate the NF-κB pathway, upregulating NLRP3 expression and forming a positive feedback loop. Meanwhile, oxidative stress in the inflammatory microenvironment can exacerbate ferroptosis. The lipid peroxides released by ferroptotic cells themselves are potent DAMPs, which in turn activate the NLRP3 inflammasome, further amplifying the inflammatory response.

It is noteworthy that the regulation of this interaction network exhibits significant dependency on cellular context. Taking the PI3K-Akt pathway as an example: in neurons, activation of this pathway tends to drive NF-κB-mediated pro-inflammatory cascades and NLRP3-mediated pyroptosis downstream [67]. In contrast, in microglia, this pathway maintains metabolic homeostasis through the mTOR axis, and its downregulation accelerates the ferroptosis process [73]. This high degree of cellular context dependency provides a fundamental rationale for understanding the phenotypic differences observed across different studies involving this pathway and underscores the need for future therapeutic strategies to account for cell type heterogeneity.

Therefore, in the face of such a complex death regulatory network, simply blocking a single pathway may not achieve ideal results, as cells can switch to other death pathways through these interaction mechanisms. Future treatment interventions should shift from “single-target blockade” to “systemic homeostasis regulation,” focusing on restoring mitochondrial function and redox balance, enhancing autophagy clearance, and synergistically inhibiting key nodes of pyroptosis and ferroptosis, thereby achieving comprehensive intervention across multiple cell death pathways and fundamentally breaking the vicious cycle of neuroinflammation.

## Potential intervention targets and treatment strategies

Modern treatment strategies for stroke are trending towards multi-target integration. Based on the key pathophysiology mechanisms and signaling pathways, numerous experimental and clinical studies have explored various treatment strategies, some of which have demonstrated certain efficacy [58]. In recent years, advancements in multi-omics technologies, along with innovative clinical trial designs, have significantly advanced the development of stroke treatment strategies [77, 78]. At the forefront of stroke treatment, precision therapy systems are evolving from acute interventions to long-term repairs, with three major innovative strategies at their focus: first, by using gene therapy to specifically silence key pro-inflammatory mediators [79]; second, by regulating the immune microenvironment through mesenchymal stem cell transplantation [80]; and third, using nano-carriers for targeted drug delivery [81]. These strategies are driving revolutionary progress in neuroprotection and repair. In this section, we take a systematic look at the current research on potential intervention targets and treatment strategies for stroke, with a focus on innovative mechanisms and their potential for clinical translation (Table 1).

**Table 1:** Potential intervention targets and treatment strategies in neuroinflammation of stroke

Intervention strategy	Specific molecule	Study Model / Design	Primary mechanism of action	Ref
Anti-HMGB1 antibodies	Anti-HMGB1 mAb (clone #10–22)	MCAO 120 min	Protects the blood-brain barrier (BBB) and effectively clears HMGB1.	[82]
	Anti-HMGB1 mAb (clone #10–22)	MCAO 120 min	Protects the BBB, alleviates brain edema, and inhibits neuroinflammation.	[83]
TLR4 inhibitors	ApTOLL	MCAO 60 min	Reduces edema, neurological deficits, hemorrhage risk, and immune responses.	[84]
NLRP3 inhibitors	MCC950	-	Reduces pro-inflammatory markers and neuronal apoptosis.	[85]
IL-1 receptor antagonists	Anakinra	A Randomized Controlled Phase 2 Trial	Reduces post-stroke plasma inflammatory markers.	[86]
MMP9 inhibitors	MMP-9 mAb (L13)	MCAO 60 min	Protects the BBB.	[87]
	MMP-9 specific siRNA/shRNA	MCAO 120 min	Protects the BBB and reduces cerebral infarct volume, brain water content, and neurobehavioral deficits.	[88]
PPARγ agonists	Rosiglitazone	MCAO 60 min	Promotes M2 polarization of microglia and reduces brain tissue loss.	[89]
Dual AMPK/Nrf2 activators	HP-1c	tMCAO	Promotes M2 polarization of microglia, exerting anti-inflammatory, antioxidant, and neuroprotective effects.	[90]
CSF-1R inhibitors	PLX5622	pMCAO	Induces microglial depletion, exacerbating infarct volume via enhanced neutrophil infiltration.	[91]
	PLX5622	MCAO 30 min	Transiently suppresses inflammation in the infarct region.	[92]
Adeno-associated virus(AAV)	AAV-shNLRP3	MCAO 60 min	Attenuates microglial pyroptosis and improves neurological recovery after cerebral ischemia.	[93]
	AAV-HRE-shCKLF1	MCAO 60 min	Inhibits neutrophil migration and infiltration, alleviating ischemic brain injury.	[94]
	AAV-PRMT8	MCAO 90 min	Promotes M2 polarization of microglia, inhibiting neuronal apoptosis and neuroinflammation.	[95]
	AAV-PHP.eB-NeuroD1-GFP	MCAO 40 min	Preserves BBB integrity, alleviating neuroinflammation and inhibiting neuronal apoptosis.	[96]
Non-viral vectors	AP@R	MCAO	Promotes the transformation of astrocytes to the A2 phenotype, mitigating neuroinflammation and facilitating BBB repair.	[97]
	siNLRP3@YWFCNPs	MCAO 90 min	Inhibits NLRP3 inflammasome activation, alleviating the inflammatory response.	[98]
Mesenchymal stem cells(MSCs)	mMSCs	MCAO 60 min	Mediates immunomodulation.	[99]
	BM-MSCs/AD-MSCs	pMCAO	Reduces cell death and improves neurological recovery.	[100]
	MSC-EVs	ICH	Modulates ferroptosis, exerting neuroprotective effects.	[101]
	microRNA-223-3p	MCAO 90 min	Inhibits M1 polarization of microglia, alleviating neuroinflammation.	[102]
	TAT&RVG-Exo + miR-15b-5p inhibitor	MCAO 60 min	Activates the HTR2C-ERK signaling pathway, inhibiting neuronal apoptosis.	[103]
Neural stem cells(NSCs)	Sbno1-NSCs-sEV	MCAO 60 min	Inhibits the MAPK/NF-κB signaling pathway, alleviating neuroinflammation.	[104]
Induced pluripotent stem cells(iPSCs)	iPS-FG	MCAO 60 min	Reduces pro-inflammatory mediator levels in the ischemic area.	[105]
	hiPSC-NPCs	MCAO 90 min	Alleviates neuroinflammation and increases the population of endogenous NSCs.	[106]
Lipid nanoparticles(LNPs)	circSCMH1@LNP1	PT 5 min	Promotes synaptic plasticity and vascular repair, alleviating neuroinflammation.	[107]
	T7-LNP@YHV984	tMCAO	Induces M2 polarization of microglia, mitigating neuroinflammation and promoting neuronal survival.	[108]
Micelles	CPLB/RAPA micelles	tMCAO	Scavenges ROS and modulates the stress signaling pathway, improving cerebral blood flow perfusion.	[109]
Exosomes	CMC-EXPL	MCAO	Drives M2 polarization of microglia, alleviating neuroinflammation and neuronal injury.	[110]

### Targeting the DAMPs-PRRs axis

In the whole process of “initiation-amplification-diffusion” in neuroinflammation following a stroke event, the DAMPs-PRRs axis occupies a central role and is an important underlying cause of neural injury and functional impairment [111]. At present, targeting upstream DAMPs with anti-HMGB1 antibodies is still in the preclinical or early clinical stages. Initially, Zhang et al. found that the intravenous injection of anti-HMGB1 monoclonal antibodies can inhibit the development of brain edema by protecting the BBB and effectively clearing HMGB1, making it an effective treatment for cerebral ischemia [82]. In subsequent animal models, the use of anti-HMGB1 monoclonal antibodies to neutralize its activity showed promise for treating stroke, effectively reducing inflammatory responses and tissue damage [83, 112]. Even with positive preclinical data, moving to human clinical applications still has to tackle issues like how well large molecules are delivered and getting past the BBB [113, 114]. Additionally, there have been some early studies on TLR4 inhibitors for treating ischemic stroke [115, 116]. Recent studies have highlighted that ApTOLL, a TLR4 modulator aptamer, had neuroprotective effects in a permanent ischemic stroke mouse model and has shown safety and efficacy in early clinical trials, whereas the specifics of clinical translation still need to be elaborated [84].

In the DAMPs-PRRs axis, the NLRP3 inhibitor (MCC950) acts as a selective small molecule inhibitor that has shown promising results in preclinical studies; however, its pharmacokinetics and toxicokinetics limit its use in clinical settings, and breakthroughs are still needed for clinical translation [85, 117]. In contrast, the IL-1 receptor antagonist (Anakinra) directly blocks inflammatory effects and has successfully completed Phase II clinical trials, as it effectively reduces post-stroke inflammatory markers and its safety in stroke patients has been confirmed [86], making it a leading drug target in clinical translation. A recent systematic review also showed that IL-1RA has potential therapeutic benefits in stroke patients [118].

Overall, targeting the DAMPs-PRRs axis is crucial for managing post-stroke neuroinflammation. Due to the complexity of stroke pathology, combining these immunomodulators with reperfusion therapy is an important focus for future trial designs.

### MMP inhibitors

In the pathophysiological process of stroke, the excessive activation of MMPs (especially MMP-9) is a key process resulting in secondary brain injury, and blocking this process is an effective method to reduce brain swelling after a stroke and protect the brain [88]. For this reason, developing drugs that target MMPs has been a major focus in this area of treatment. However, the many failed clinical trials over the years indicate that the clinical efficacy of broad-spectrum MMP inhibitors is limited [119]. Therefore, using highly selective MMP inhibitors is seen as the most promising direction for research and development in this area.

Currently, various biological therapeutic strategies are being explored to achieve the above goal. Early studies have shown that MMP-9 neutralizing monoclonal antibodies can reduce the size of infarcts in animal models by specifically binding to and inhibiting MMP-9 activity [120]. Recent research has also found that MMP-9 neutralizing monoclonal antibodies can reduce BBB disruption in stroke mice [87]. Gene silencing strategies, using viral vectors or nanoparticles (NPs), like quantum dots and gold NPs, have also been employed to deliver MMP-9 specific siRNA/shRNA, effectively downregulating its expression at the genetic level. This therapy has shown that targeting MMP-9 can help ease the effects of ischemic stroke and improve outcomes [88]. Additionally, the antibiotic minocycline has become a unique and well-regarded drug for treating strokes due to its multifunctional neuroprotective mechanisms and good safety profile. It is noteworthy that multiple preclinical studies have confirmed its neuroprotective effects. However, a phase IV clinical trial (NeuMAST) for AIS did not achieve its primary efficacy endpoint, and the interim analysis was terminated early due to futility [121]. The excessively broad therapeutic time window used in this trial and patient heterogeneity in stroke may be important reasons for the negative results. The pleiotropic effects of minocycline as an antibiotic present both advantages and challenges; its mechanism of action involves multiple pathways such as MMPs, apoptosis, and inflammation, but this multi-target nature makes it particularly challenging to precisely define its therapeutic window and applicable population [122].

### Regulation of glial cell polarization

Neuroinflammation after stroke is a key contributor to secondary brain injury, and regulating the polarization of microglia/macrophages towards an anti-inflammatory repair type has become a prominent method for alleviating inflammation and promoting neural repair. Scholars are indeed paying increasing attention to peroxisome proliferator-activated receptors (PPARs) in neuroinflammatory diseases because of their anti-inflammatory and neuroprotective effects [123]. Studies have shown that PPARγ agonists (rosiglitazone) help keep white matter healthy in the long run after a stroke by driving microglia to polarize towards the anti-inflammatory M2 type [89]. However, metabolic effects of PPARγ activation, including insulin sensitization, may increase glucose fluctuations during the acute phase of stroke, thereby undermining its clinical feasibility for intervention at this stage. Additionally, metabolic side effects such as weight gain and edema further complicate clinical management [124]. In addition, Nrf2, as a key player in regulating oxidative stress, directly impacts the extent of damage and the recovery outlook after stroke [125]. Research by Wang et al. found that dual AMPK/Nrf2 activators indeed help cut down on post-stroke brain inflammation by enhancing microglial M2 type polarization [90].

It is worth noting that in the treatment of stroke, how precisely microglia are regulated by colony-stimulating factor-1 receptor (CSF-1R) inhibitors is highly dependent on the timing of treatment. Research has found that the continuous administration of PLX5622 before and after the middle cerebral artery occlusion (MCAO) model causes microglial depletion, which not only increases neutrophil infiltration but also makes the infarct area bigger [91]. Another study showed that although the long-term use of PLX5622 can temporarily suppress inflammation in the infarct area, it also disrupts microglial balance and the tissue reperfusion process, emphasizing the importance of treatment timing [92].

In summary, the strategies discussed so far aim to turn the harmful inflammation after a stroke into a healing immune response. To this end, strictly controlling the treatment time window is key to making this transformation happen while also protecting the brain and keeping harmful inflammation at bay.

### Other strategies

At the frontier field of stroke treatment, three major directions are pointing to the precision medicine era: gene therapy is aimed at precisely regulating the expression of inflammation-related genes; stem cell therapy works by regulating the immune system and repairing nerves; and nanodrug delivery systems allow for the targeted delivery of anti-inflammatory drugs through the BBB. Together, these strategies provide a promising new approach for protecting and repairing the nervous system.

#### Gene therapy

The initiation of neuroinflammation in stroke involves complex molecular mechanisms. Relevant research on gene therapy is still in the early stages, and most studies are still in the preclinical phase. Viral and non-viral vectors are key delivery methods in gene therapy. Among the various viral vectors, adeno-associated virus (AAV) is especially suitable for gene therapy in the central nervous system because it provides stable gene expression and has high safety, making it an important option in this area [126]. On the one hand, AAV can be used to inhibit key mediators of neuroinflammation and change the inflammatory environment. Studies have found that knocking down the NLRP3 inflammasome with AAV can reduce microglial pyroptosis, enhance white matter integrity, and improve neurological recovery after cerebral ischemia [93]. Similarly, using AAV vectors guided by HIF-1α to deliver shRNA that knocks down CKLF1 in the ischemic area can significantly reduce the migration and infiltration of neutrophils, which helps lessen brain injury from ischemia in rats [94]. On the other hand, AAV can also help shift microglial cells to the anti-inflammatory M2 type. Research has shown that in an ischemic stroke model, the level of PRMT8 reduces significantly, while injecting AAV-PRMT8 can boost Lin28a levels, thereby helping microglial cells to become similar to the M2 type and effectively reducing neuronal apoptosis and neuroinflammation [95]. Furthermore, AAV also shows promise in keeping the BBB intact. He et al. found that injecting the AAV-PHP.eB vector with NeuroD1 can help repair blood vessel damage and neuron loss after a stroke, offering new ideas for gene therapy in stroke treatment [96].

Although viral vectors have demonstrated high efficiency in gene delivery, their clinical application is often limited by significant side effects, including immunogenicity, potential carcinogenic risks, and poor target cell specificity. As a result, non-viral vectors are increasingly seen as a more feasible alternative strategy in gene therapy [127]. In recent years, the integration of nanotechnology with gene therapy methods has provided new possibilities for overcoming the BBB, enabling the safe and efficient delivery of gene therapies to brain lesion areas [128]. A recent study found that polymer NPs loaded with Rolipram (AP@R) effectively inhibited Lcn2 expression, helping to reduce neuroinflammation and repair the BBB [97]. Another study developed a long-circulating near-infrared II (NIR-II) nanoparticle platform (YWFC NPs) that boosts transcytosis through the BBB. In a preclinical model of ischemic stroke, this platform successfully delivered a targeted NLRP3 siRNA (siNLRP3@YWFC NPs), effectively inhibiting NLRP3 inflammasome activation and significantly alleviating the inflammatory response [98].

Although the aforementioned strategies have shown promising efficacy in preclinical models, no gene therapy targeting neuroinflammation in stroke has been approved to date. This is largely due to the disparities between laboratory and clinical settings: First, most existing studies utilize young, healthy mouse models, whereas stroke patients are predominantly elderly and often have comorbidities such as hypertension and diabetes. These factors alter BBB permeability and metabolic states, resulting in much lower vector delivery efficiency in humans than anticipated. Second, gene therapy typically requires several days to weeks to reach peak expression, creating a temporal mismatch with the treatment window during the acute ischemic phase. Finally, the potential off-target effects of gene-editing tools may cause irreversible genomic damage, which is the primary concern for regulatory agencies when approving entry into clinical trials. In summary, gene therapy offers a revolutionary tool for intervening in post-stroke neuroinflammation. However, to achieve clinical translation, future research should focus on developing humanized disease models, optimizing vector targeting capabilities, and establishing rigorous off-target assessment systems.

#### Stem cell therapy

Stem cell therapy, as an emerging strategy, has shown great promise in managing neuroinflammation after stroke. Studies show that, in the context of the specific animal intracerebral hemorrhage (ICH) model, stem cell therapy can regulate the immune microenvironment and promote the recovery of neurological function [129]. Instead of directly replacing neurons; instead, it adjusts the immune environment with strong paracrine effects, alleviating the inflammatory response after stroke and promoting the recovery of neurological function [130].

Recently, mesenchymal stem cells (MSCs) have become the preferred cell type in clinical research due to their low immunogenicity, great immunoregulatory abilities and good safety profile [131]. For instance, the intravenous infusion of mouse bone marrow-derived syngeneic MSCs (mMSCs) have achieved immunomodulatory functions in experimental stroke [99]. Subsequent experiments in rats confirmed that the intravenous injection of donor-derived bone marrow MSCs (BM-MSCs) or adipose-derived MSCs (AD-MSCs) can reduce cell death, help with cell proliferation, and improve neurological recovery [100]. Further research revealed that the benefits of MSCs are partly due to the extracellular vesicles (EVs) they release [132]. A recent study found that MSC-EVs can protect neurons from damage caused by ICH via regulating ferroptosis [101]. In addition, MSC-derived exosomes (MSC-Exos), given their immunoregulatory and tissue regeneration properties, have become a promising option for treating serious central nervous system injuries, achieving neuroprotective effects in various injury models including stroke [133]. Moreover, overexpressing microRNA-223-3p in MSC exosomes can help reduce cerebral I/R injury by inhibiting the polarization of microglia to the M1 phenotype and its mediated pro-inflammatory response [102]. Another recent study found that MSC exosomes modified with TAT and RVG can effectively deliver microRNA-15b-5p inhibitors, which can prevent neuronal death caused by cerebral ischemia-reperfusion via activating the HTR2C-ERK signaling pathway [103]. In summary, several phase II clinical trials have tentatively confirmed the efficacy and safety of MSC therapy; however, researchers still need to figure out the best treatment timing and long-term effectiveness of this treatment modality.

Neural stem cells (NSCs) possess the ability to differentiate into neurons, astrocytes and oligodendrocytes, making them a promising target in stroke research [134]. In terms of endogenous repair, physical regulation methods such as rTMS have been shown to promote the proliferation and differentiation of endogenous NSCs, providing a cellular basis for the reconstruction of damaged neural networks [135]. This process is closely related to the regulation of neurotrophic factors. For example, BDNF can effectively promote the proliferation and differentiation of NSCs. Moreover, this effect is positively correlated with the volume of ICH [136]. In terms of exogenous treatment, recent studies have confirmed that NSCs that overexpress strawberry notch 1 (Sbno1) secrete Sbno1-NSC-derived small EVs (sEVs), which help reduce neuroinflammation following cerebral ischemia by inhibiting the MAPK and NF-κB signaling pathways in microglia, significantly promoting recovery from ischemic stroke [104]. Although NSCs have the potential to directly differentiate into neural cells, the source of these cells and the risk of immune rejection are holding back their clinical application. To tackle these issues, the emergence of induced pluripotent stem cell (iPSC) technology offers a great alternative. This technology turns differentiated somatic cells back into a pluripotent state through gene reprogramming, and the resulting iPSCs possess advantages such as self-renewal, multipotent differentiation, and low immunogenicity, making it an indeed appealing new therapeutic strategy [137]. Early studies have shown that in the MCAO rat model, subdural transplantation of iPS mixed with fibrin glue (iPS-FG) can effectively reduce the levels of pro-inflammatory mediators in the local ischemic area [105]. In addition, recent findings further revealed that in the same MCAO rat model, the intracerebral transplantation of neural precursor cells (NPCs) derived from human HLA-homozygous iPSCs (hiPSC-NPCs) can help boost the body’s natural repair process, which shows up as reduced neuroinflammation and more endogenous NSCs [106]. Overall, iPSCs are still in their early days in clinical research, and their long-term safety (especially the risk of tumorigenesis) is still a significant challenge in advancing clinical applications.

Although stem cell therapy holds great promise, its clinical translation still faces significant challenges. Firstly, because of their large size, stem cells are highly susceptible to extensive retention in the lungs during intravenous administration due to the “pulmonary first-pass effect,” resulting in extremely low efficiency in targeting brain lesions [138]. Secondly, donor variability and limited in vitro passage numbers cause significant batch-to-batch differences in cell preparations, directly affecting the consistency of efficacy in clinical trials [139]. Additionally, the risks of tumorigenicity from the long-term survival of stem cells in vivo and issues of ectopic tissue formation are major obstacles in regulatory approval. The core of future development lies in creating biomaterial-assisted “off-the-shelf” universal cell products to enhance cell colonization efficiency and overcome heterogeneity. However, whether these strategies can ultimately bridge the clinical translation gap still depends on the dual challenges of cell-specific barriers and personalized stratification strategies in synergistic application.

#### Nanoparticle drug delivery systems

In recent years, nanoparticle drug delivery systems have attracted considerable attention because of their unique physical and chemical properties. On the one hand, they can precisely target lesions; on the other hand, they can effectively load and deliver drugs that regulate neuroinflammation, offering new strategies for treating strokes. Currently, various nanoparticle delivery platforms such as liposomes, micelles, polymer nanoparticles (PNPs), and exosomes are in the development stage. By delivering therapeutic drugs just where they are needed in the ischemic penumbra, these systems aim to reduce nerve damage, help repair nerves, and ultimately improve the outlook for stroke patients [140].

Lipid nanoparticles (LNPs), given their excellent biocompatibility and high modifiability, have been widely used in the treatment of brain diseases such as stroke, neurodegenerative diseases, as well as gliomas and other brain tumors. The surface functionalization of LNPs based on antibodies or peptides not only improves the efficiency of BBB penetration and achieves precise and targeted drug delivery but also directly exerts multiple therapeutic functions such as anti-inflammation, tissue repair and neuroprotection [141, 142]. Recent studies have established that intranasal delivery of LNPs loaded with circSCMH1 (circSCMH1@LNP1) significantly increases their distribution in the area around the infarct, thereby promoting synaptic plasticity, aiding vascular repair, and alleviating neuroinflammation, ultimately improving sensory-motor and cognitive functions in the photothrombotic (PT) stroke model mice [107]. Another study developed T7-targeted peptide-modified LNPs to deliver YHV984. This nanoparticle (T7-LNP@YHV984) effectively alleviated neuroinflammation and promoted neuronal survival by inhibiting the Hv1/NLRP3 pathway and inducing microglia polarization towards the M2 phenotype, ultimately boosting survival rates and neurological recovery in mice [108]. These findings offer new directions and add to the experimental evidence for developing LNP-based neuroprotective strategies. Research has also shown that micelles possess advantages such as improving BBB penetration efficiency, high effective payload, and targeted T cell delivery, allowing for the precise control of T cell function, thereby reducing immunosuppression and enhancing stroke efficacy [143]. A polymeric micelle system (CREKA-PEG-LysB/Rapamycin, designated CPLB/RAPA) with neurovascular targeting and mTOR inhibition achieves controlled drug release. This system effectively clears ROS and helps regulate stress signaling pathways, improving cerebral blood flow perfusion by remodeling the neurovascular unit [109], which was found to be a viable target for nanoparticle therapy in ischemic stroke.

It is worth noting that a large number of polymers can be used to form various nanocarrier systems, which can be divided into two main categories based on their source: natural and synthetic. PNPs, as a prevalent platform, have demonstrated significant advantages in stability and drug-loading capacity [144]. Exosomes, as nanoscale membrane vesicles from endosomes, play a key role in intercellular communication by delivering functional components such as mRNA, miRNA and proteins to recipient cells. Their inherent stability, low immunogenicity, and ability to efficiently penetrate the BBB make them a promising alternative for brain repair and the treatment of ischemic brain conditions [145]. Recent research has even developed a functionalized poly(lactic-co-glycolic acid) (PLGA) nanomedicine called CMC-EXPL based on macrophage-derived exosomes for the targeted co-delivery of dual drugs. This system drives the reprogramming of microglial cell phenotypes from pro-inflammatory M1 to anti-inflammatory M2, effectively alleviating neuroinflammation and neuronal injury in rat model studies [110]. This study demonstrated how nanomedicines work together in vivo, highlighting the huge potential of dual-drug combination strategies.

Despite the significant achievements in preclinical studies of nanomedicines, their clinical translation still faces distinct challenges. Firstly, the complexity of the nano-bio interface results in reduced efficacy. After entering the bloodstream, NPs form a protein corona on their surface, which not only masks targeting ligands but also makes them prone to clearance by the immune system. This results in accumulation in the liver and spleen and potential toxicity risks [146]. Additionally, insufficient assessment of long-term degradation kinetics and neurotoxicity in the brain has led regulatory agencies to adopt a cautious approach to approval. Future breakthroughs will depend on simplifying designs to improve production feasibility, such as developing biomimetic nanoplatforms or personalized cell delivery systems to circumvent the protein corona effect.

## Future directions and prospects

Going forward, exploring the mechanisms that initiate neuroinflammation in stroke is expected to shift the main research focus towards a deep understanding and careful control of the spatiotemporal heterogeneity of neuroinflammation, which is essentially a complex process that changes over time with the disease course. Regarding the acute phase of stroke, studies will focus on how to effectively curb the excessive activation of microglia and peripheral infiltrating immune cells to reduce further damage. Future efforts should look into the dynamic changes of inflammatory signaling pathways during the acute phase and find effective means to regulate them to alleviate the acute breakdown of the BBB and neuronal injury. As stroke progresses to the chronic phase, the challenge for researchers will shift to how to actively terminate persistent chronic inflammation and reprogram the neuroimmune environment, changing it from blocking repair to supporting new nerve growth and axon remodeling. Getting this balance right between acute protection and chronic repair is crucial for improving the long-term outlook for stroke patients.

However, bringing this research perspective to life faces fundamental challenges at the level of translational medicine. Currently, the strong results obtained from standardized animal models are often difficult to replicate successfully in highly heterogeneous clinical patients, which highlights the challenges of translating findings due to species differences and the complexity of diseases. In the future, we need to create models that better reflect human physiology and pathology by constructing animal models that take into account human genetics and mimic the effects of comorbidities, or by using cutting-edge technologies such as human organoids and organ-on-a-chip to better mimic the complex human cerebrovascular and immune environments in the lab. Additionally, tools such as imaging and fluid biomarkers should be utilized to classify inflammation in clinical trials. On this basis, individualized treatment strategies should be developed, tailoring treatment plans based on each patient’s specific inflammatory status, genetic background and other traits, to avoid differences in treatment effectiveness due to patient diversity. It is also crucial to strengthen interdisciplinary collaboration and improve clinical trial design, as this will help us systematically evaluate how different strategies work at various inflammatory stages and among different patient groups, an essential aspect for effectively translating basic discoveries into clinical applications.

In addition, single-cell sequencing and multi-omics integrated analysis jointly constitute a key driver of development of this field. Single-cell sequencing technology analyzes the heterogeneity of various cell types involved in neuroinflammation, accurately identifying key cell subpopulations such as pro-inflammatory and anti-inflammatory cells and revealing their unique molecular characteristics and functions, which provides targets for precise cellular interventions. Building on this, multi-omics integrated analysis combines genomics, transcriptomics, proteomics, and metabolomics data to systematically build a dynamic regulatory network for neuroinflammation, identifying key molecular markers and signaling pathways related to disease progression and treatment response, thereby moving treatment approaches closer to precision medicine. The deep integration of single-cell and multi-omics technologies lays the foundation for a new era of precision stroke treatment. Meanwhile, utilizing artificial intelligence algorithms to integrate multimodal data and establish predictive models can identify individuals at high risk for neuroinflammation early and help develop smart diagnostic and treatment systems, thus offering real-time, dynamic data support for clinical decisions, ultimately creating a complete feedback loop from scientific discovery to clinical practice.

In summary, the main future direction of research on neuroinflammation in stroke involves analyzing the temporal and spatial differences, breaking down barriers in translational medicine, and using advanced technologies for personalized treatment. Through multidisciplinary collaboration and technological innovation, it is hoped that “precision anti-inflammation” can be achieved that protects nerve function, helps with recovery, and significantly improves patient outcomes.

## Data Availability

Not applicable.

## Abbreviations

DAMPs: Damage-associated molecular patterns
PRRs: Pattern recognition receptors
BBB: Blood-brain barrier
ATP: Adenosine triphosphate
HMGB1: High mobility group box 1
HSPs: Heat shock proteins
IL-1β: Interleukin-1beta
TLRs: Toll-like receptors
NF-κB: Nuclear factor-kappaB
TNF-α: Tumor necrosis factor-alpha
ROS: Reactive oxygen species
BDNF: Brain-derived neurotrophic factor
GDNF: Glial cell line-derived neurotrophic factor
XO: Xanthine oxidase
MMPs: Matrix metalloproteinases
RNS: Reactive nitrogen species
NETs: Neutrophil extracellular traps
Treg: Regulatory T cells
CKLF1: Chemokine-like factor 1
OGD/Re: Oxygen-glucose deprivation/reoxygenation
CPT1A: Carnitine palmitoyltransferase 1 A
GTA: Glyceryl triacetate
CMPK2: Cytidine/uridine monophosphate kinase 2
AIS: Acute ischemic stroke
BCAA: Branched-chain amino acid
DHI: Danhong injection
ASS1: Argininosuccinate synthase 1
PRDX1: Peroxiredoxin-1
PCD: Programmed cell death
MCAO: Middle cerebral artery occlusion
STING: Stimulator of interferon genes
rTMS: Repetitive transcranial magnetic stimulation
THBS1: Thrombospondin-1
PPARs: Peroxisome proliferator-activated receptors
AAV: Adeno-associated virus
MSCs: Mesenchymal stem cells
EVs: Extracellular vesicles
ICH: Intracerebral hemorrhage
NSCs: Neural stem cells
iPSC: Induced pluripotent stem cell
LNPs: Lipid nanoparticles
